# Socio-cultural profile of Bapedi traditional healers as indigenous knowledge custodians and conservation partners in the Blouberg area, Limpopo Province, South Africa

**DOI:** 10.1186/s13002-015-0025-3

**Published:** 2015-06-06

**Authors:** Malehu K Mathibela, Bronwyn A Egan, Helena J Du Plessis, Martin J Potgieter

**Affiliations:** Department of Biodiversity, School of Molecular and Life Sciences, University of Limpopo, Private Bag X 1106, Sovenga, 0727 South Africa

**Keywords:** Blouberg area, Socio-cultural profile, Traditional healers, Indigenous Knowledge, Community conservation

## Abstract

**Background:**

Bapedi traditional healers of Blouberg are custodians of indigenous knowledge on medicinal plants of this region. They provide primary health care to a large number of people in the Blouberg area of South Africa. There is concern that this profession is dying out, which may be detrimental to the Blouberg community and to biodiversity conservation in the area.

**Methods:**

Thirty two healers and 30 community members were interviewed between March 2011 and July 2013 around Blouberg Mountain in the Blouberg Municipality. A semi-structured questionnaire was used to elucidate socio-cultural and demographic variables and healing customs of practicing healers. Attitudes to sustainable management of medicinal plants were captured. A second semi-structured questionnaire was used to gather information on community members’ views of traditional healers and their practices.

**Results:**

Sixty seven percent of interviewed community members visited traditional healers. Female traditional healers dominated (80%) the profession. Sixty four percent of the healers have no formal education, with only 4% having secondary school education. Seventy nine percent of healers see between 15 and 20 patients per month. Clinics and a hospital in the vicinity have resulted in a shift by the community from using tradition-based healing to that of allopathic health care. Most interviewed traditional healers (71%) are in favour of conservation actions to prevent over-harvesting, as 86% believe that indiscriminate collecting is compromising the flora of the area. Most (93%) are willing to use cultivated plants.

**Conclusions:**

State health care has negatively influenced the practice of traditional healing as patients now first consult government health centres before turning to traditional healers. In the past, traditional healing has been ignored because, as an oral history, it could not be included in school curricula or government policy documents. Those traditional healers who learn to write will have the skills to document and safeguard their own knowledge. This can help to prevent the erosion of knowledge around Blouberg’s medicinal plants and support the conservation of natural resources in the area. Adult learning programmes might therefore be worth implementing amongst healers.

## Background

On a world-wide scale, indigenous knowledge, including that related to traditional healing, is seen as waning [1]. At the same time and paradoxically, global demand for traditional healing plants is growing [2]. In Africa, the situation is similar. Despite evidence that over 72% of the African population use traditional medicine as their first choice when seeking health care [3] and that trade in medicinal plants is growing rapidly [4], some studies report that the custodians and practitioners of this knowledge are becoming fewer. [5]. Peltzer [6], for example, reports in a survey of South African traditional healing, that use of traditional healers has decreased in recent years. Furthermore, in a broad study which examined health care choices in South Africa, Grobler and Stuart [7] noted a general decline in the use of traditional healers amongst South Africans.

Two reasons for this inconsistency are that recruitment to the profession of traditional healing is difficult to measure [8] and that precise data, using both qualitative and quantitative methods, are lacking [6]. Traditional medicines are now readily available to the public from muthi shops, which are African chemists selling traditional medicines for curing physical, spiritual and cultural ailments [9], as well as from traders. This means that people can self-medicate without requiring the intervention of a trained traditional healer [10]. This leads to the anomaly that although traditional medicines are generally preferred and used by most Africans [11], the healers themselves complain that they cannot rely on the proceeds of their healing practice alone for their livelihoods, and emerging healers are few [8].

Traditional healers in Africa may be decreasing, but they are still consulted on a wide scale and are readily available [12], affordable and trusted [13]. State-sponsored primary health care systems in Africa are limited and between 70% and 80% of the rural population lack access to these institutes [14]. Where they are available, the quality is poor, thus people resort to visiting traditional healers who are still present in most communities [15]. Furthermore, patients appreciate the holistic nature of a traditional healer’s curation approach, which attends to disorders of both mind and body [16]. Traditional healers are socially acceptable to communities as they are of the same culture and are therefore more effective than allopathic practitioners at treating culture-based health problems [12]. They are thus frequently the first health care providers to be consulted for bodily illnesses as well as spiritual matters [17].

In the rural Blouberg area of the Limpopo Province, South Africa, traditional healers [18,19] as well as a state sponsored hospital and various clinics [20] are available. Limited research has, however, been conducted on the importance of traditional healing to the communities in Blouberg and none on the socio cultural profile of these healers. It is not clear, therefore, whether traditional healing is a dying practice in this area, nor what the community’s attitude is towards their different health care options. Semenya and Potgieter [19] conducted an overall study of the traditional healers of Limpopo and recommended further research with larger sample sizes for each municipality in order to verify their findings. This Blouberg study is therefore a response to the suggestion. Considering that in a review of the use of complimentary health practitioners in South Africa, Peltzer [6] called for more research into the concurrent use of traditional health practitioners and allopathic health care, the Blouberg situation is of interest.

Allied to the concern that traditional healers are declining, is the recognition that on a global scale, medicinal plants are also rapidly dwindling [2]. At the same time there is a world-wide surge of interest in these plants [1] as evidenced by the recent publication of handbooks on medicinal plants and reviews on globally important medicinal species [21-23]. In Africa, commercial interest, as well as a growing population, many of whom use traditional medicinal plants, has resulted in a sharp increase in harvesting of these plants [24]. In the past, traditional healers would themselves collect the plants they required for their healing practices. There were rituals and traditions associated with this collection, which, to a certain extent, protected the plants from over-harvesting, either by limiting the amount of material removed or by restricting the type of harvesting such that damaging procedures were restricted [25]. With the growth of the “muthi” (medicinal) plant industry in Africa, commercial harvesters have come to dominate the collection of medicinal plants and rituals and traditions are not observed [26]. These collectors are not closely associated with the environments from which the plants are sourced and do not have an interest in sustainable harvesting. If the resource base collapses in one area, they then source plants from another. The result is over-harvesting, which is exacerbated by a weak legislative framework governing the use of plant resources and inadequate environmental law enforcement [27].

Within the Blouberg area, the Blouberg Mountain is a rich repository of medicinal plants and home to declining species such as *Boophane disticha* (L.f.) Herb. and *Hypoxis hemerocallidea* Fisch., C.A. Mey. & Avé-Lall., as well as endangered species such as *Warburgia salutaris* (Bertol.f.) Chiov. [28]. This is not surprising as the mountain is part of the Soutpansberg Centre of Endemism [29], and has recently been defined as a Critical Biodiversity Area in the Limpopo Provincial Conservation Plan 2013. Vegetation types include endangered grassland, fynbos, savanna, sourveld and forest elements, each of which support a different suite of medicinal plants [18]. Despite the conservation importance of the Blouberg Mountain, it is currently not protected.

Natural resources in general are under threat and there is a call for including the public more strongly in developing policies for the protection of the natural environment [30]. The presence of people in a community who value their local natural resources affords these ecosystems some informal protection [31]. People who are knowledgeable about the uses of local biodiversity and are already organized into a group which functions towards common goals make valuable contributions towards protecting local natural areas [32,10].

The traditional healers of Blouberg are an example of such a group. Blouberg Mountain is under threat of transformation by the mining sector as well as by the development of housing and roads [33]. The healers in the area have an interest in conserving the natural plant resources close to them and although they would harvest the plants, this could be more efficiently managed for biodiversity conservation than the overall transformation that mining or large scale infrastructure development would bring [34]. Indeed, Hamilton [2] emphasises that conservationists who are interested in protecting medicinal plants should never do so in isolation from the people who use them. As he further explains, conservationists need to have an understanding of the human communities that are dependent on the plants, including the culture, economics and social structure of the relevant society. An understanding of the socio-cultural profile and demographics of the Blouberg traditional healers would thus be useful to conservationists managing the Blouberg Mountain. Understanding the traditional healing sector in this area will give insight into how such people may already be safeguarding the natural resources in Blouberg, and how they might do so more effectively in the future.

In the context of this discussion, the broad term “traditional healer” is used. This term generally includes herbalists and diviners [35]. Herbalists are those who work mainly with the application of herbal remedies [16]. They diagnose the illness of a patient through careful observation and discussion with the patient and prescribe plant-based medicines for everyday ailments. In contrast, diviners diagnose primarily via spiritual means and serve as important intermediaries between humans and the supernatural [11].

Both herbalists and diviners are present in the Blouberg area; however, most traditional healers define themselves as herbalists, whereas only a minority are seen as diviners. During meetings, Blouberg traditional healers stressed that both herbalists and diviners in the area use medicinal plants collected from the Blouberg Mountain [18]. Furthermore, Richter [36] stated that a strict separation between traditional healers and diviners is becoming more difficult in modern times. For these reasons, no distinction is made between herbalists and diviners in this paper and they are referred to interchangeably as traditional healers.

This study contributes to the dialogue on traditional healers by investigating the demographics and socio-cultural profile of Blouberg’s traditional healers, as well as the attitudes of the Blouberg community to traditional healing. It clarifies whether or not the practice is disappearing in Blouberg and relates this to the scenario in Africa as a whole. The potential of the traditional healers to act as custodians of their natural environment is discussed, as is the impact on the environment of a decline in traditional healers and associated knowledge.

## Methods

### Study area and study population

**Figure 1:**
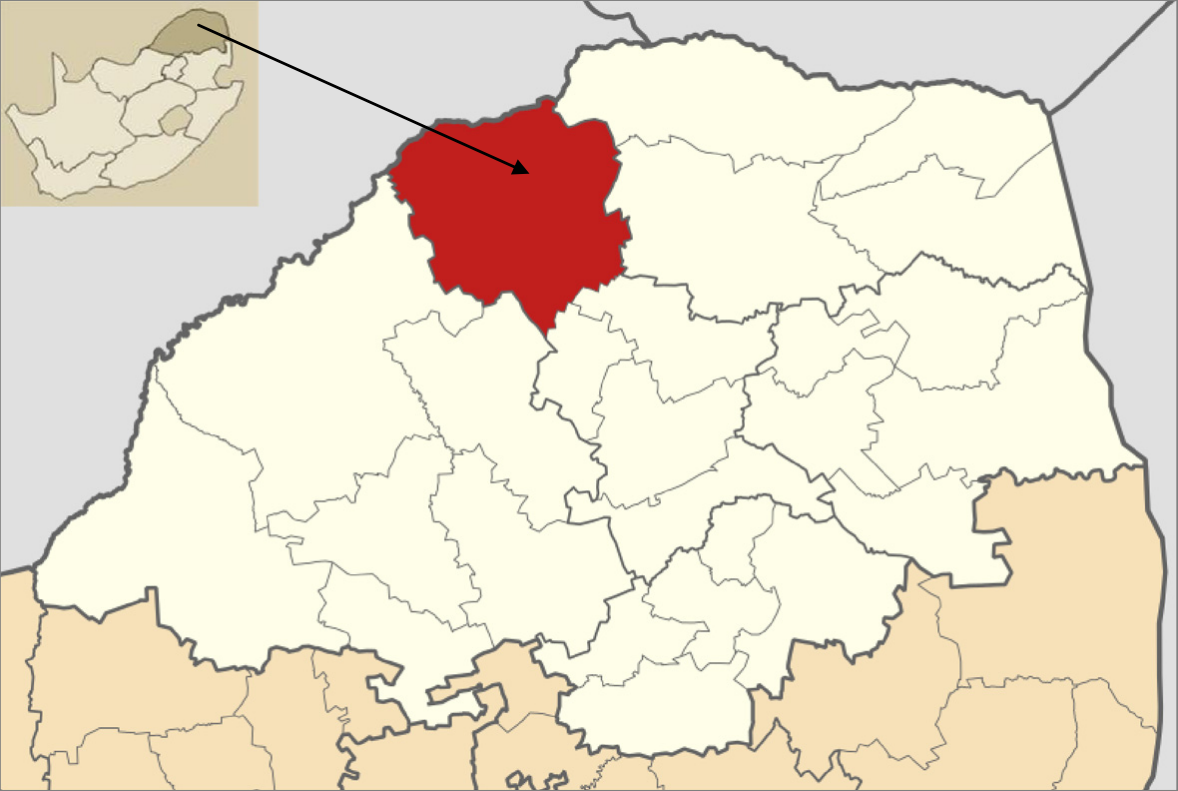
Location of the Blouberg area, Limpopo province, South Africa.

The study was conducted around the Blouberg Mountain in the Blouberg area, Limpopo Province, South Africa (Figure 1). The municipality covers an area of approximately 5 054 km^2^ and stretches up to the Botswana border [33]. The study was, however, restricted to the area on and around the Blouberg Mountain in order to ensure that healers interviewed were using the mountain as their closest source of medicinal plants.

Sixteen villages (Table 1) were selected from around Blouberg Mountain. Villages further away were not used, as there would then be the possibility that people residing in these villages do not use the mountain as a source of medicinal plants. Face-to-face meetings were held with various local groups of traditional healers. The reasons for the meetings were to introduce the project, to determine how active traditional healers were in the area and to enlist them for the study. Ultimately, 32 healers from 16 villages volunteered to participate. The traditional healers interviewed were of the Bapedi tribe as they are the dominant cultural group in the study area. In the Blouberg study area, 90 traditional healers are registered with the local traditional healers association and although there are others who are not registered, these were not included in the study due to the difficulty of locating them.

**Table 1:** **Villages around Blouberg Mountain from which traditional healers were selected**

A	Blackhill	E	Bull Bull	I	Springfield	M	Kwarung
B	Inveraan	F	Varedig	J	Sweethome	N	Bosehla
C	Bobeng	G	Stockings	K	Liepsig	O	Dantzig
D	Normandy	H	Glenfernest	L	Mafateng	P	The Glade

Using the snowball sampling technique [37], 30 community members were drawn from the same villages as those of the healers. They were interviewed to determine the attitudes of the general population to the different choices available for healing and in particular the importance of traditional healers in maintaining their good health.

Participants gave their informed consent for the publication of all results and any accompanying images before commencing with the interview schedules as required by the University of Limpopo’s ethics committee. Random sampling could therefore not be undertaken as it is a requirement that participants volunteer to be included in the study. This could have introduced some bias in that only those people with enough time to be interviewed, and/or those interested in the study volunteered. Conducting shorter interviews with fewer questions would have allowed for a larger sample size but at the expense of the depth and quality of the data. As this was the first study of its kind in the Blouberg area, the focus was on depth to gain more insight into the situation and in future, larger sample sizes will be selected in order to be able to generalize more fully.

### Data collection

Data was collected between March and July 2013 using two semi-structured questionnaires. The first questionnaire was designed for the healers in order to gather data on socio-cultural issues. These included: (i) Gender and (ii) level of education, (iii) questions dealing with traditional practices: Age at becoming a healer and years of experience, (iv) reasons for taking up the trade, (v) number of patients, (vi) apprentices and (vii) attitudes towards conservation of medicinal plants.

The second semi-structured questionnaire was designed for the community members in order to capture the following information: (i) health care options chosen and reasons for these choices, (ii) advantages of consulting traditional healers over state health facilities, (iii) ailments treated, and (iv) level of satisfaction with the traditional healers’ services.

Data obtained from both questionnaires were analysed by means of descriptive statistics.

## Results and discussion

### Traditional healers

#### Gender

The traditional healing profession in the Blouberg area is dominated (80%) by females. This dominance is not unique to the Bapedi as similar findings were reported for the Zulus in South Africa by De Wet et al. [38], for the Baka in Cameroon [39], the Masai in Kenya [40] and further afield in Brazil [41]. However, for the Bapedi in general, this female dominance is unusual. Both Moeng and Potgieter [42] and Semenya [43] noted the dominance of Bapedi males in traditional healing in the Limpopo Province of South Africa. The 2011 population census found that in the Capricorn District (which includes Blouberg Municipality), from the age of 25, females dominate the population [20]. In the age bracket 65–69, which is the age of most Blouberg traditional healers, females in the Capricorn District dominate by almost a third. This trend is also true for the Limpopo Province in general. For the Blouberg Municipality specifically, Census (2011) [44] stated that females outnumber males by almost one fifth. Although this population trend must influence the ratio of female to male healers, it does not, however, explain the extreme dominance of 80% of female traditional healers, nor why Blouberg should differ from the general trend of gender ratios amongst Bapedi traditional healers in the rest of the Limpopo Province.

Voeks [41] noted that women from Lenḉois community in Bahia, Brazil, were more knowledgeable than men in identifying and naming useful medicinal plant species. In addition, women, rather than men, are the custodians of Lenḉois medicinal plant knowledge. Another study in Ethiopia also revealed that it is local women, not men, who are the most knowledgeable about plants which provide medicinal and veterinary services [45]. Women are generally the caregivers in society, medicating and nursing family members when they are ill [46], therefore their broader knowledge of medicinal plants is understandable. Knowledge and interest in medicinal plants might prompt more women than men to follow the calling of traditional healing.

Many useful medicinal plants are located in and around homesteads such as in home gardens, pastures and croplands, as well as within disturbed areas [47]. It is women who are involved in maintaining these areas and therefore women who come into most contact with these medicinal plants. Since prehistoric times, men tend to work further afield, in the pre-industrial era to hunt, and latterly, employed as migrant workers away from home [48,41]. They are therefore less intimately involved with local natural resources and less likely to be able to learn as much as women regarding their uses. This is true of the Blouberg area, which is regarded as one of the least developed regions in the Limpopo Province, with the lowest level of income and highest unemployment rate (90%) in the country [33]. These poor socio-economic conditions result in large numbers of young men leaving the area in search of migratory jobs. Men are more likely than women to work in the mines, farms and industries, which are further away from the Blouberg area. These factors may help to explain female dominance in this trade, but do not explain why the traditional healing fraternity in Blouberg area should differ from the rest of Limpopo in terms of gender ratios, as these socio-economic conditions predominate in most rural villages in Limpopo where healers are generally male.

### Education

This study found that 64% of traditional healers in the Blouberg area have no formal education, 32% attended primary school and only 4% attained secondary school level. In their more widespread study of healers from Limpopo Province in general, Semenya and Potgieter [19] reported similar findings, with 95% of male and 77% of female healers having no, or only rudimentary, schooling. The long apprenticeship that Blouberg healers usually serve negatively influences their education level. This is in line with findings of Mthembu [49] who reported that 40% of Zulu healers at Umlazi Township in KwaZulu-Natal, South Africa, had no formal education. However, findings by Gessler et al. [11] showed that these low levels of education are not universal in Africa, with 61% of the healers in Tanzania managing to reach primary school and only 23% with no formal education. The fact that the majority of healers in South Africa have no formal education as opposed to those in Tanzania, cannot be blamed solely on socio-economic conditions, as both countries are classified as developing. Thus, it would seem that other factors also play a role in the low level of education of traditional healers in South Africa, especially in the Blouberg area. Possibly this has to do with the poor quality of education in South Africa and rural areas in particular [50].

Adult Basic Education and Training should be made available to those traditional healers who feel that such courses will be to their benefit. Reading, writing and arithmetic are useful tools for business people, thus traditional healers who are proficient at using such tools may find this impacting positively on their income. The ability to write will give the healers the opportunity to document and disseminate their indigenous knowledge themselves, independently of outsiders’ input and bias and at their own discretion. Communication skills will enhance their ability to collaborate with institutes of research and technology, enabling them to benefit further from their indigenous healing knowledge through publications and intellectual property safeguards. Full participation in procedures and policies to protect the environment (e.g., environmental impact assessments) is also reliant on literacy. Compliance with environmental legislation relating to the collection of medicinal plants is facilitated by being able to read. In the past, literacy level has not negatively affected the success of the traditional healing profession, however, lately, information technology is penetrating even remote rural areas through cell phones (mobiles) and computers [51]. Healers who are able to keep in touch with clients via instant messaging through cell phones will be at an advantage over those who cannot through lack of reading skills.

### Age at becoming a healer, and experience or years in the trade

Healers around Blouberg area entered the profession at the young age of between 18 and 23 years old. The future of traditional healing in the Blouberg area is dependent on younger people filling the ranks, however, half of the healers (50%) in Blouberg are elderly, (over 60 years of age) and younger people are not being trained, as evidenced by the fact that only 3% of healers are less than 35 years of age. Forty seven percent of the healers are between the ages of 50 and 60. This might be due to the fact that most young people in Blouberg relocate to escape the prevailing high levels of poverty [52]. Even if they later become healers, they might not service the Blouberg area. In this study the largest age class was also the oldest, which indicates a declining body of traditional healers in the Blouberg that will ultimately result in a loss of indigenous knowledge.

The years of experience that healers have in the Blouberg area reflect the age structure. Twenty nine percent of the healers have less than 10 years of experience and 21% have between 11 and 20 years of experience. Seven percent of respondents have between 21 and 30 years of healing experience, while 43% have more than 30 years of experience in health practice.

### Reasons for taking up the trade

Reasons for entering the trade relate to following a parental tradition and being called upon by ancestors. This calling is usually by means of a dream. It is customary that the ancestors select someone within a family who possesses the necessary potential for healing. This scenario is common to many African traditional healers. For example, according to Moagi [53], the person is shown, through their dreams, a place where they must go to be trained. In Blouberg, apprentices serve under an established healer for a number of years, as also occurs elsewhere in Africa [42,54].

### Patient numbers

Only seven percent of the respondents see more than 30 patients per month. Another 7% see between 21 and 30 patients per month and most respondents (79%) see between 15 and 20 patients per month. A minority of 7% do not see a single patient in a month. The healers noted their concern about the low number (average 15 per month) of patients that consult them. This is in contrast to traditional healers in Tanzania who see between 1 and 200 patients per week [11]. Reasons put forth by Blouberg healers for the low number of consultations relate to the large number of traditional healers in the area, the uneven use of healers due to favouritism, and the presence of a number of clinics and a hospital in the area.

Although traditional healers charge a very low consultation fee, the provision of state-sponsored, free primary health care in the Blouberg area has had a devastating effect on traditional healing as a profession. The availability of a hospital and clinics in the Blouberg area is an attempt by the South African government to improve the well-being of people in this area. Kaplan [55] noted that a shift from using traditional healers to consulting western physicians occurs with socio-economic upliftment, and ultimately results in culture change. Muela et al. [56] stated that in Tanzania, people resorted to traditional healers only when they felt that the treatments they received at local hospitals were not effective or could not treat their culture-based illness. Due to the factors mentioned above, some healers in the Blouberg area are considering closing their practices.

### Apprentices and continuity of healing in Blouberg

The vast majority (82%) of Blouberg traditional healers do not use apprentices for collecting plant materials, as they believe that they best know the locality of certain plants, as well as when and how to perform certain rituals before collecting them. However, according to Tshisikhawe [57], Venda traditional healers from South Africa train their apprentices so that they may be as good as traditional healers at collecting plant material. Thus when healers are away on collection trips, apprentices are able to stand in for them thus preventing a loss of income via consultation fees. The practice of using apprentices, who may then continue the traditional healing trade, may be related to the monetary benefits attained by the healers. Healers in Ghana, as long ago as 1987, lamented the fact that they could not persuade young people to enter the profession because they themselves could not make a living from traditional healing alone and needed to supplement their income. Young people therefore prefer to move to urban centres and search for work in other more conventional fields where they will be remunerated at a higher level [8]. In Blouberg, the traditional healers do not generally take on alternative work, despite being the main breadwinners in their families. Many of the female healers are also widows. They see their work as a sacred calling and despite the fact that patient numbers are few and income modest, they usually do not supplement their income. Thus, as in Ghana, young people are not attracted to this calling.

### Attitude to conservation management of medicinal plant collection

Seventy one percent of traditional healers would be satisfied if conservation authorities or the Traditional Healers Association enforced restrictions on plant collecting to reduce overharvesting. The 22% who were not in favour of harvest restrictions were of the opinion that it would then be a waste of time to go up the mountain to collect the small amount of plant material allowed. Seven percent felt indifferent.

**Figure 2:**
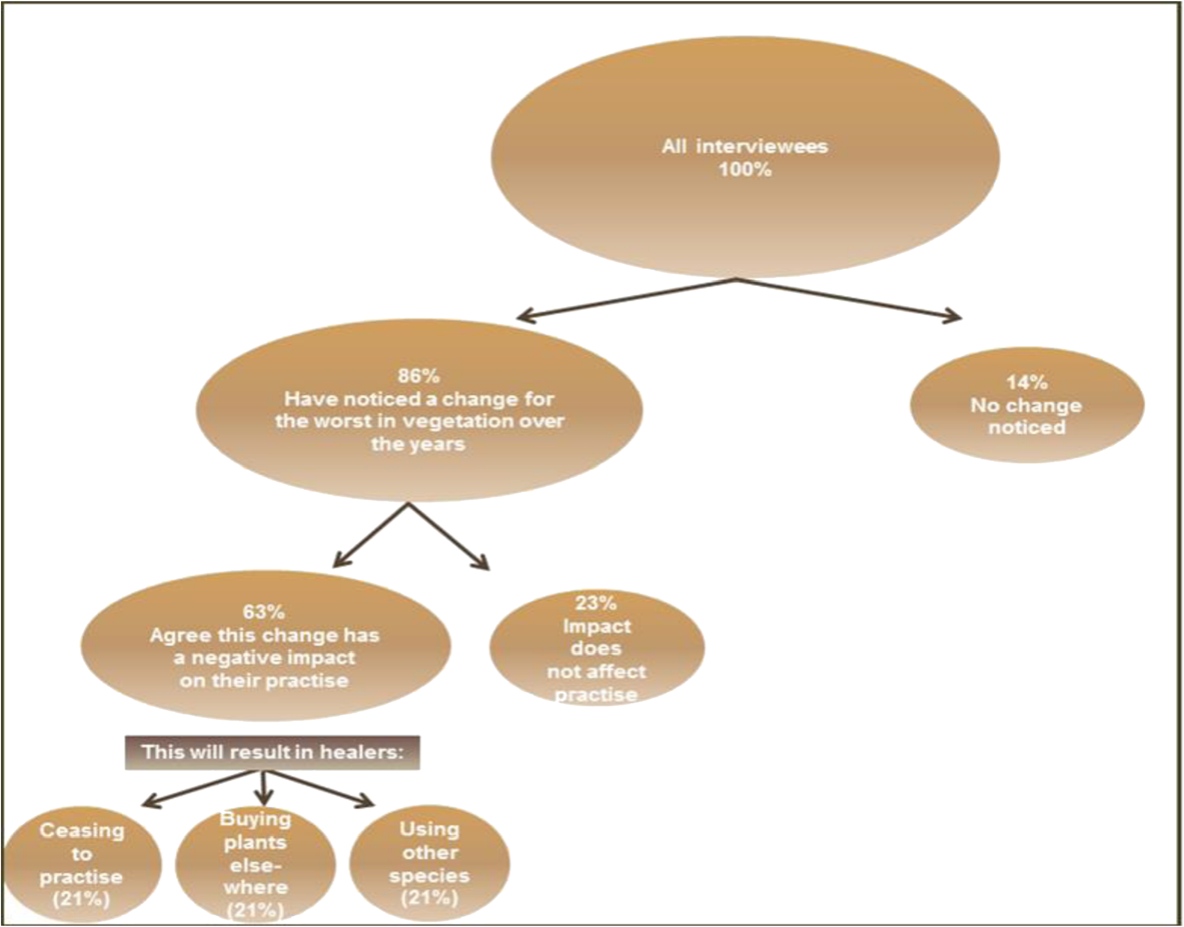
Attitudes that Blouberg Mountain traditional healers have regarding changes in vegetation on the mountain over time and their attitudes thereto.

Eighty six percent of the interviewees have noticed a change for the worse in the vegetation on the mountain over time. Attitudes to this change are captured in Figure 2. This indicates that Blouberg traditional healers are very much aware of the decline in the quality of natural resources on the Blouberg Mountain and are unhappy about this fact. Most of them are willing to take steps to remedy this even though it will result in harvesting limits.

Ninety three percent of the interviewed traditional healers would use cultivated plants if available and would even appreciate a nursery in their area. The minority who would not use cultivated plants believe that these plants are less powerful than wild grown ones. Eleven percent of the healers have home gardens where a number of species are grown. This is a further indication of the effort that the traditional healers are prepared to make to conserve these important plants.

### Community members

#### Health care options chosen and reasons for these choices

Health care options for the people of Blouberg Municipality include allopathic, state sponsored and private doctors, traditional healers and Christian faith healing. The government provides one hospital, two health care centres and 23 clinics for the Blouberg Municipality [20].

Despite these choices, the majority (67%) of the community members around Blouberg Mountain who were interviewed consult traditional healers. This is slightly lower than the estimated 80% of the Zulu population in South Africa that visit traditional healers [58]. In a country such as South Africa, where access to medical doctors is limited, traditional healing plays an important role [59] and this seems to be true for the Blouberg area too. Of those respondents that consult traditional healers, 95% also visit state sponsored health facilities such as clinics and a hospital. The 5% that consult only traditional healers note that they trust the healers’ judgment and can rely totally on the healers for obtaining help.

Fifty five percent of those who call on traditional healers indicated that the regularity of their consultations over the past five years have remained the same. Twenty percent noted that their visits have declined in number because they have recovered and not become ill again. The remaining quarter mentioned that their visits have increased.

Most people in rural areas consult traditional healers for a wide range of physical, psychological and social problems. According to Abbo [60], people in Africa have a close relationship with their traditional healer who is often from the same community and culture. This is also the case in Blouberg where the majority of the respondents consult traditional healers for the following reasons: (i) ill health (ii) bewitchment, (iii) bad luck, and/or (iv) disturbing dreams.

Although rural people rely on traditional medicine because it is cheap, locally available and easily accessible, not every person in rural areas consults traditional healers [8]. This is also true for the Blouberg area where 33% percent of respondents do not make use of traditional healers at all. Of these respondents, 40% avoid healers because they are Christians, and according to their religion, this is forbidden and they make use of state sponsored health facilities. Ten percent of those who do not consult healers rely only on spiritual healing from their church when they are ill. Thirty percent give no reasons for avoiding healers, but 10% stated that they do not trust them to heal their ailments. Another 10% visit only hospitals for their health care needs.

Anyinam [8] found that the changing beliefs and values of the people; the introduction of Western institutions such as education, allopathic medicine, and Christianity; the transition from a rural subsistence lifestyle to one based on a monetary as well as the changing lifestyle of the people, all contributed to a decline in traditional healing in Ghana. Inheritance of the knowledge from parent to children is also becoming more infrequent for the same reasons. These factors are relevant in the Blouberg setting where Christianity is becoming more widespread and people are less influenced by ancestral worship. Education is also more attainable and a subsistence lifestyle is becoming more and more difficult with the influence of climate change. The increased accessibility of shops as well as clinics and a hospital practicing allopathic medicine have all ensured that the people of Blouberg are less influenced by a traditional way of life that places high emphasis on traditional medicine and are more inclined to follow both systems of healing.

### Advantages of consulting traditional healers over state health facilities

Community members gave several reasons for choosing traditional healers over state health care professionals: (i) No improvement in health after visiting state facilities, (ii) state doctors do not explain ailments or the cause of problems in as much detail as healers, and (iii) healers are more readily available than conventional doctors and have fewer and shorter waiting lines. Indeed, 70% of respondents believe that traditional healers are more holistic in their approach to health care, 20% believe that traditional healers obtain better results in curing ailments, with 5% stating that they are cheaper than state health facilities and 5% mentioning the shorter waiting lines for traditional healers. In a nation-wide South African survey of those who use traditional healers, Grobler and Stuart [7] reported similar reasons for consulting healers. In particular, the fact that treatment was effective was most important, as is the case for Blouberg. Respectful and holistic treatment and shorter waiting lines were also valued, as found in this study.

### Ailments treated

Blouberg respondents who consult traditional healers prefer a more inclusive service and wish to understand emotional and spiritual aspects of their lives, as well as the material ailments they suffer from. They would like to have a better understanding of the meaning of what is happening in their lives currently and what is going to happen in the future. In the opinion of the Blouberg respondents, the state-employed allopathic doctors cannot answer these questions satisfactorily. These reasons are in line with those given by Robertson [61], in a South African review paper. He stated that many people in rural areas view traditional healers as providing more holistic care than western medical practitioners and as having an approach that is more acceptable.

Patients further believe that there is a lack of knowledge by western medical practitioners in the treatment of culture-bound syndromes, and that there are some ailments that can only be treated by traditional medicine. For example, mental or spiritual illnesses may be treated by a traditional healer using traditional medicine, while gastrointestinal disorders may be treated by a western physician [62]. The community in the Blouberg area believes that state health care professionals may treat illnesses such as headaches, toothache, allergies, chest pains, flu as well as testing for HIV/AIDS.

Despite the present use of traditional healers in the Blouberg area and the positive references to choosing traditional healers when seeking health care, Western medicine has begun to replace traditional medicine in the Blouberg area. This has resulted in a gradual decline in the service provided by traditional healers in treating bodily illnesses. On the other hand, traditional healers are still consulted widely when illnesses are thought to have a spiritual origin or patients are concerned about their mental wellbeing.

### Level of satisfaction with the traditional healers’ services

Of those respondents who consult traditional healers, 45% mentioned that their problems were resolved after one visit, whereas another 50% noted that their problems were not resolved and that they had to consult again before they recovered. For 5% their problems were never resolved. In contrast to our findings, a study by Ayibor [63] in South Africa found that the majority of community members (75%) indicated that their children recovered well after visiting a traditional healer. Their ailments were cured and they expressed their willingness to visit traditional healers again. Twenty-five percent indicated that their children’s conditions worsened, and they required further consultations or had to resort to visiting a hospital. In Blouberg area, about half the patients of traditional healers are satisfied with the results they obtain. There is thus room for improvement and this may be one reason for low patient numbers in Blouberg.

### Insights into traditional healing, loss of indigenous knowledge and links to conservation

Blouberg healers are predominantly women who start their healing profession at a young age. They have rudimentary formal education and do not see large numbers of patients when compared with those in the rest of Africa. They collect their own plants, rejecting the use of apprentices who may not know the correct places to collect plants or the rituals that should be performed whilst collecting them. Half of the healers are over 60 years of age, with only 3% under 35 years of age and the practice is therefore dying. This dwindling group of women has custodianship of a valuable source of local information about the Blouberg healing plants, but their knowledge is vanishing before its value is truly known. Custodianship implies not just knowledge but also an understanding of the value of that knowledge as well as a willingness to ensure that it is used wisely and safeguarded [64]. According to Maila and Loubser [65], there are three groups of custodians of indigenous knowledge. The first are intellectuals who are intent on integrating indigenous knowledge with other knowledge to find solutions for African needs. The second are rural practitioners of indigenous knowledge who have little or no formal education, but who use indigenous knowledge in their daily lives in order to solve every day problems. They pass this knowledge on to apprentices or to their children. The third group are urban indigenous knowledge holders, who, during their life engagement, create and use modernised traditional knowledge. In the Blouberg area, the women who practice traditional healing fall into the second group as they have an extensive knowledge of medicinal plants and also safeguard this knowledge through their membership of the Blouberg Traditional Healers’ Association [18]. There is wide acknowledgement that it is important to include women’s local and traditional knowledge in managing for biodiversity [66], however, as Momsen [48] laments, few women with such knowledge are involved with government-level environmental decisions. This is certainly true for the Blouberg area.

Xu et al. [67] stated that supporting indigenous cultures supports the conservation of biodiversity. This could well be done on a small scale in the Blouberg area by capacitating the healers through further education and training programmes, particularly in aspects of environmental legislation. This would provide them with the skills to contribute to environmental debates within their local municipality or through their traditional leader.

## Conclusions

Findings from this paper indicate that although the majority of Blouberg residents still consult traditional healers and are satisfied with the service provided, the practice is slowly declining and the related knowledge of healing plants is in danger of being lost. The lack of recruitment of younger healers and the presence of state sponsored health facilities have contributed to this decline. Acknowledging the value of the wisdom these healers possess and documenting important aspects with sensitivity and an awareness of intellectual property rights is a priority in supporting the healers.

Scientific validation of the efficacy of herbal treatments and assisting in the documentation of aspects of this traditional knowledge will support responsible traditional healing in the Blouberg area. Empowering traditional healers to participate in local environmental planning would be valuable in light of the fact that local people’s points of view are frequently ignored during mining and development applications. Ultimately, supporting the traditional healing culture, preserving the associated knowledge and using this to promote conservation in Blouberg, would be the responsibility of the community itself. Scientists and government conservation agencies need, however, to provide the momentum for such actions by disseminating relevant research findings through community workshops. All these actions will be facilitated by supporting further education and training programmes amongst traditional healers. This will give them the tools to enable them to document and disseminate their important body of indigenous knowledge themselves.
